# Genetic Differentiation among *Maruca vitrata* F. (Lepidoptera: Crambidae) Populations on Cultivated Cowpea and Wild Host Plants: Implications for Insect Resistance Management and Biological Control Strategies

**DOI:** 10.1371/journal.pone.0092072

**Published:** 2014-03-19

**Authors:** Tolulope A. Agunbiade, Brad S. Coates, Benjamin Datinon, Rousseau Djouaka, Weilin Sun, Manuele Tamò, Barry R. Pittendrigh

**Affiliations:** 1 Department of Entomology, University of Illinois at Urbana-Champaign, Urbana, Illinois, United States of America; 2 USDA–ARS, Corn Insects and Crop Genetics Research Unit, Ames, Iowa, United States of America; 3 International Institute of Tropical Agriculture, Cotonou, Benin; Zhejiang University, China

## Abstract

*Maruca vitrata* Fabricius (Lepidoptera: Crambidae) is a polyphagous insect pest that feeds on a variety of leguminous plants in the tropics and subtropics. The contribution of host-associated genetic variation on population structure was investigated using analysis of mitochondrial cytochrome oxidase 1 (*cox1*) sequence and microsatellite marker data from *M*. *vitrata* collected from cultivated cowpea (*Vigna unguiculata* L. Walp.), and alternative host plants *Pueraria phaseoloides* (Roxb.) Benth. var. *javanica* (Benth.) Baker, *Loncocarpus sericeus* (Poir), and *Tephrosia candida* (Roxb.). Analyses of microsatellite data revealed a significant global *F_ST_* estimate of 0.05 (*P*≤0.001). The program STRUCTURE estimated 2 genotypic clusters (co-ancestries) on the four host plants across 3 geographic locations, but little geographic variation was predicted among genotypes from different geographic locations using analysis of molecular variance (AMOVA; among group variation −0.68%) or *F*-statistics (*F*_ST_ *^Loc^* = −0.01; *P* = 0.62). These results were corroborated by mitochondrial haplotype data (*φ_ST_^Loc^* = 0.05; *P* = 0.92). In contrast, genotypes obtained from different host plants showed low but significant levels of genetic variation (*F*_ST_^Host^ = 0.04; *P* = 0.01), which accounted for 4.08% of the total genetic variation, but was not congruent with mitochondrial haplotype analyses (*φ_ST_^Host^* = 0.06; *P* = 0.27). Variation among host plants at a location and host plants among locations showed no consistent evidence for *M. vitrata* population subdivision. These results suggest that host plants do not significantly influence the genetic structure of *M. vitrata*, and this has implications for biocontrol agent releases as well as insecticide resistance management (IRM) for *M. vitrata* in West Africa.

## Introduction

Host plant adaptation by herbivorous insects has resulted in monophagous species that are highly specialized on a single host, whereas polyphagous insect species have evolved to feed upon a wide array of different host plants (e.g., [1]–[3]). Host plants may have a major role in the differentiation and diversification of herbivorous insects, and are important in our current understanding of global biodiversity and niche exploitation by insect populations [4]–[6]. The diversity of ecosystems, which polyphagous species encounter, makes the study of genetic variation based on host plants important for the understanding of adaptation and niche formation. Within a single species, genetic variation can arise among subpopulations that utilize different host plants through variation in oviposition or feeding preferences, rates of development on different host plants, as well as subsequent survivorship, fecundity and mating preferences of adults [7]. Mating barriers and reduced gene flow have been predicted among individuals from insect species that show adaptation to different host plants [8]–[9], and resulted in assortative mating within populations [10]. In addition to broader implications in species formation, assortative mating based on host plant preference can impact the practical application of insect pest management strategies, such as the release of biocontrol agents and the implementation of insect resistance management (IRM) strategies based on genetically modified crops.

The legume pod borer, *Maruca vitrata* Fabricius (Lepidoptera: Crambidae) is a polyphagous insect pest of grain legumes that has a wide distribution throughout tropical and subtropical regions worldwide. Feeding damage caused by larval *M. vitrata* to cowpea crops occurs on flower buds, flowers and seed pods. This insect species develops without diapause and uses multiple alternative host plants during the dry season in West Africa when cowpea crops are not in cultivation [11]–[13]. Larval *M. vitrata* feeding has been documented on over 50 alternative host plants [11], [13]–[14], and most often found on cultivated and wild host plants from the family, Fabaceae [15]–[16]. *Pterocarpus santalinoides* L'Hér. ex DC., *P. phaseoloides* and *Centrosema pubescens* (except cv. Belalto) are used for oviposition and subsequent larval development during the long dry season, whereas *Lonchocarpus sericeus* and *L. cyanescens* (Schumach and Thonn.) Benth. are similarly used during the main rainy season, and *Tephrosia platycarpa* Guill. and Perr. during the short rainy season [13]. The reservoirs of *M. vitrata* maintained on alternative host plants results in difficulties for cultural and chemical insecticides control. As proposed by [17], the possible Asian origin of *M. vitrata* may contribute to the lack of corresponding native natural enemies capable of regulating its populations in those alternative host plant habitats in West Africa, and thus might also lead to heavy infestations observed on cowpea crops. Efforts to introduce biological control candidate species have had limited success, and yet unrecognized biotic factors such as *M. vitrata* alternative host plant differentiation, could hinder the effective spread of introduced control agents [18].

Protein crystalline (Cry) toxins produced by the gram-positive soil bacterium *Bacillus thuringiensis* (*Bt*) show insecticidal activities against many Lepidopteran insects. Transgenic cowpea that express the *Bt* toxin Cry1Ab are being developed for the protection of this crop for use in West African cropping systems [19]. Although transgenic *Bt*-cowpea offers a promising approach to crop improvement, sustainability of the technology will likely depend on the mitigation of resistance development in *M. vitrata* populations and availability of suitable alternative host plants to act as refuges. Specifically, the high-dose refuge model is the most widely accepted IRM strategy [20], and has been implemented as an effective resistance management plan to delay the development of resistance to *Bt* in target pest insect populations [21]. The high-dose component of this IRM strategy requires that crops express levels of *Bt* toxin sufficient to kill 100% of homozygous susceptible and heterozygous larvae. Refuges are non-*Bt* plants in proximity to *Bt* crops on which the targeted pests can also complete development [21]. In theory, refuge plants are able to produce a large population of adults that will mate randomly with any potential homozygous resistant individual that might complete development on a *Bt* crop plant. By shear stochastic sampling, rare homozygous resistant individuals that emerge from *Bt* fields are most likely to mate with a refuge plant-derived homozygous susceptible individual. This increases the probability that any resistant insects emerging from the *Bt* crops are more likely to mate with a susceptible adult emerging from the refuges, thereby generating heterozygous progeny that are not capable of surviving exposure to the high dose of *Bt* toxin expressed by transgenic crop in order to delay or prevent an increase in resistance allele frequency within target insect populations [22]. Wild-growing alternative host plants can also serve as natural refuges for target pests, and have been reported as effective refuges for IRM of transgenic crops [23]–[27]. In the case of *M. vitrata*, there are several alternative host plants which are available throughout the cowpea growing season and which might act as natural refuges. Assessing the suitability of alternative hosts as effective refuge plants for *Bt*-cowpea will be important for developing IRM programs for *M. vitrata* in West Africa. However, it is not clear when *Bt*-cowpea will be used broadly in West Africa, which highlights the need to enhance the efficacy of current pest control solutions.

The control of *M. vitrata* in West Africa currently relies on the use of cultural and chemical control methods and increasingly on the use of biological control agents. Alternative host plant use and any potential genetic differentiation among populations based on this biological phenomenon may also impact how biocontrol agents are deployed [28]. The lack of alternative hosts may be a contributing factor in the observation that, although many biological control introductions result in establishment, most are unsuccessful in reducing pest densities [29]. Therefore, most managers of agricultural systems seek to manipulate habitat complexity to encourage the conservation and enhancement of natural enemies in the hopes of improving pest suppression (see reviews by [30]–[32]). A key factor that enhances predator and parasitoid populations in complex landscapes is the availability of nectar and pollen subsidies. Many natural enemies, particularly hymenopteran parasitoids, lacewings, syrphid flies, and tachinid flies are herbivorous as adults and require carbohydrates for successful reproduction. A literature review by [33] showed that the successful establishment of certain parasitoids in cropping systems depends on the presence of weeds that provide nectar for the adult female wasps. Laboratory and field studies have also demonstrated positive impacts on parasitoid fecundity, lifespan, or searching efficiency as a result of floral resources in bordering non-crop areas [34]–[37]. However, although alternative host plants have been reported to enhance parasitoid and predator efficiency in conservation biological control strategies, extensive population-level data are still needed for deployment of biocontrol agents to be effective. The application of population genetic data to biological control of *M. vitrata* will provide better information on how many distinct genotypes exist on the different host plants and the effect this can have on the parasitoid population over time. The use of population structure data will therefore enable the identification of the genetic differentiation of *M. vitrata* on cultivated cowpea and available alternative host plants and the effective host plants that can be planted alongside the cultivated cowpea in order to maximize parasitoid efficiency.

Genetic variation among *M. vitrata* larvae on four host plants including cowpea in West Africa was assessed using haplotype sequencing of the mitochondrial cytochrome c oxidase-1 gene (*cox1*) fragment, as well as genotyping using a set of microsatellite markers previously developed by [38]. Levels of genetic and haplotype variation, population structure, and gene flow were estimated among *M. vitrata* collected from different host plants in southern regions of Benin. The results of this research are important for assessing the effectiveness of alternative host plants for use as a refuge for *Bt*-cowpea crops, and to potentially identify the most appropriate host plant to apply biocontrol agents. These data will be used to enhance ongoing efforts to reduce the impact of *M. vitrata* feeding damage and to improve yields in cowpea cropping systems of West Africa.

## Materials and Methods

### Ethics Statement

For all the insect samples used in the study, no permission was required for the insect sampling and collection. Insect sampling and collection was performed with our collaborators at the International Institute of Tropical Agriculture (IITA), Benin. Permission was not required because the insects used for the study are common insect pests on legumes, and IITA Benin has a Memorandum of Understanding (MOU) with the government of Benin for conducting research on these insect pests. The insects used for this study are not endangered species.

### Insect Sampling and DNA Extraction

**Figure 1:**
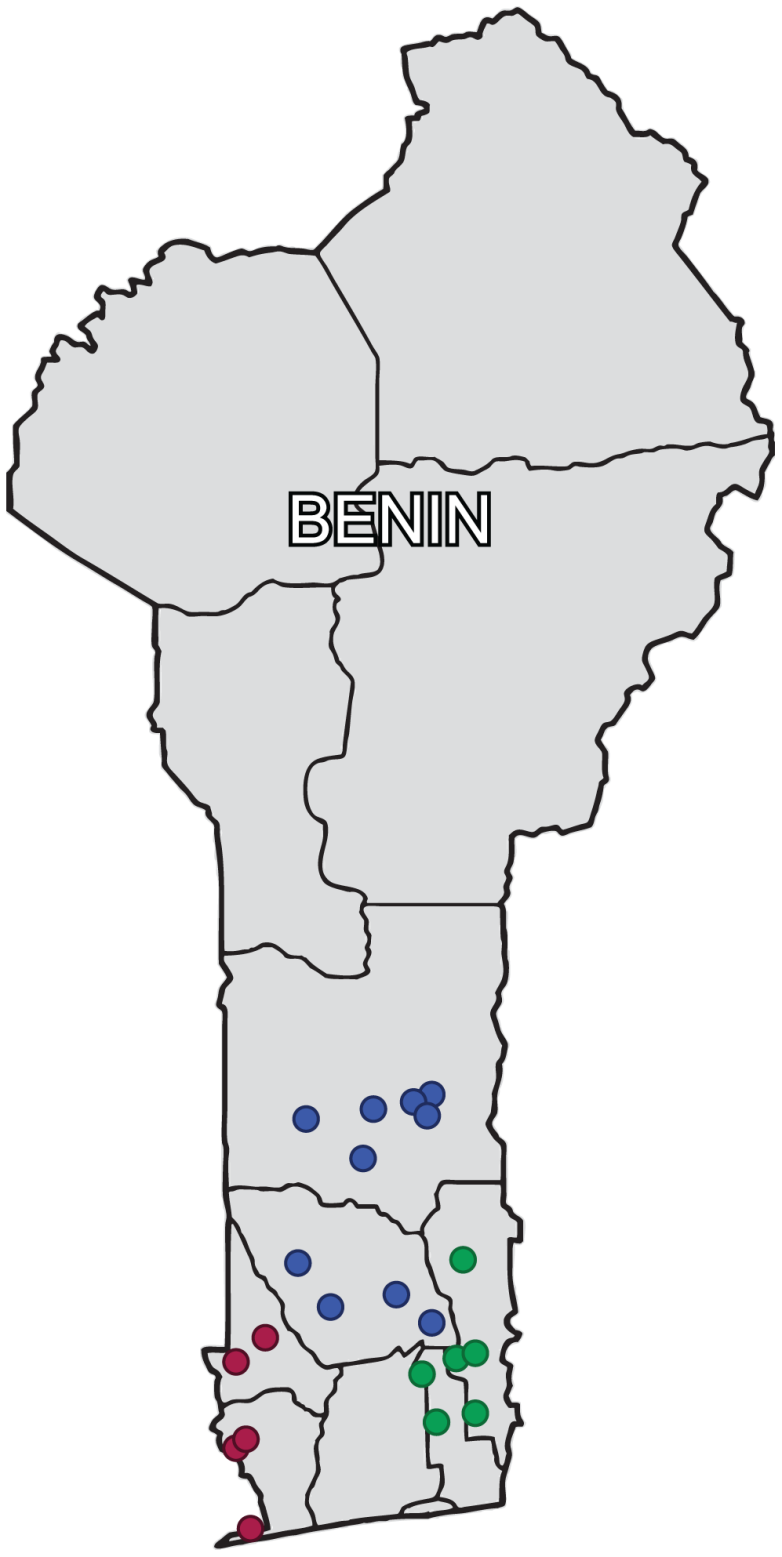
Map showing collection sites in southern Benin (red circles – Mono-Couffo, blue circles – Zou-Collines, and green circles – Ouémé-Plateau).

Larval *M. vitrata* were collected from cultivated cowpea (*Vigna unguiculata*), and three alternative host plants – *P. phaseoloides* (dry season host), *T. candida* (short rainy season host), and *L. sericeus* (main rainy season host), in three divisions representing 6 departments in Southern Benin in 2012 (Figure 1). The divisions were Mono-Couffo, Zou-Collines and Ouémé-Plateau. Within each division, we collected from different locations to lessen the possibility that the same female individual laid larvae collected. Forty-nine, 50 and 49 individual *M. vitrata* samples were collected from *V. unguiculata* in Ouémé-Plateau, Zou-Collines, and Mono-Couffo, respectively. Forty-seven and 45 individual *M. vitrata* samples were collected from *L. sericeus* in Ouémé-Plateau, and Zou-Collines, respectively. Fifty-two, 52 and 58 individual *M. vitrata* samples were collected from *T. candida* in Ouémé-Plateau, Zou-Collines, and Mono-Couffo, respectively, and 49, 49 and 48 individual *M. vitrata* samples were collected from *P. phaseoloides* in Ouémé-Plateau, Zou-Collines, and Mono-Couffo respectively. Genomic DNA was extracted from the insect samples using DNeasy animal tissue kit and following manufacturer instructions (Qiagen, Valencia, CA). The DNA concentrations were adjusted to 10 ng/μl and used for genotyping.

### Microsatellite Genotypes

Microsatellite markers C0241, 7_02K06, C0444, C32008 and 01_B12 were used for genotyping *M. vitrata* samples (Table 1), amplified in multiplex PCR reactions and detected as previously described by [38]. The microsatellite markers were obtained as previously described in [38] and the DNA sequence libraries submitted to GenBank under the accession numbers from JN685509 to JN685580. The mean number of alleles per locus, observed heterozygosity and expected heterozygosity were calculated for genotypes by location and by host plant within each location using Arlequin v3.5.1.3 [39]. The potential occurrence of null alleles and other genotyping errors (stuttering and allele drop out) were tested using the program Micro-Checker v2.2.3 [40], and null alleles were suspected at a given locus when Micro-Checker rejected Hardy-Weinberg Equilibrium (HWE) and excess homozygosity was evenly distributed among allelic size classes. Null allele-corrected pairwise *F*_ST_ estimates were calculated for all populations by applying the ENA correction in the FreeNA package ([41]–[42]; available at http://www1.montpellier.inra.fr/URLB/). Uncorrected *F*_ST_ values were estimated following [43], whereas corrected *F*_ST_ estimates were made when null allele were predicted following the expectation maximization (EM) algorithm [44].

**Table 1:** *Maruca vitrata* primer sequences used for microsatellite amplification reactions.

Locus	Primer (dye label) and sequence (5′-3′)	Repeat	Size (bp)
C32008^ E^	F-(MAX)AAAAAGCGCTTATATGTTTGTTATAGT	(CATA)_3_	163
	R-GAAATTTTTAACGGAGATACAATCA		
7_02K06^ A^	F-(FAM)ATTTGTCAGAATGGTATCTTACGT	(GAT)_6_	151
	R-CCTCTGGGTCATAATTATATTGTTCA		
C0444^ E, 1^	F-(FAM)AAAGGAACTACGCCGTCAGG	(CAA)_8_	102
	R-GTTGAGCGATCTTGGCACAG		
C0241^ E^	F-(TAM)GACGAAACAAGGCCTACCAG	(GAT)_9_	165
	R-GGTACTTCYGACGTTGTTCG		
01_B12	F—(TAM)CGGGATGTTACATATACCCAGCA	(CA)_12_	119
	R-CGTACCAATTCATTGAGACTCTCTT		

E, EST-derived primer pair; A, anonymous genomic sequence-derived primer pair; 1, PCR multiplexed primers.

Analysis of molecular variance (AMOVA), global *F*-statistics [45] and pairwise *F_ST_* estimates were calculated also using Arlequin v3.5.1.3 [39]. Four different analyses were performed based on assumed partitioning of the population based on host plant and/or geographic location; *analysis 1*: variation among host plants (pooled across all locations), *analysis 2*: variation among geographic locations (pooled for all host plants), *analysis 3*: differentiation between host plant within each geographic location, and *analysis 4*: differentiation between geographic location for each host plant group. Significance for each comparison was corrected for Type I error by application of the B-Y method [46].

The program STRUCTURE v2.3.4 uses a model-based clustering to predict population structure using genotypic marker data from individual samples, where the model assigns proportions of individual genotypes to one of *K* populations [47]. STRUCTURE analysis of microsatellite genotype data was run using an initial burn-in of 100,000 iterations followed by 100,000 iterations, and ten replicates with each potential value of *K* (range 1 to 10) were run with an assumed population admixture model. STRUCTURE runs were performed using LOCPRIOR command, where genotypes were defined based on host plant at each geographic location. The ‘real’ value of *K* (number of potential unique populations represented by the *M*. *vitrata* genotypes) was estimated as described by [48] using the program Structure harvester ([49]; available at http://taylor0.biology.ucla.edu/structureHarvester/). A graphical display of individual co-ancestry (*Q*-matrix) data was generated from STRUCTURE output using the program Distruct [50].

Isolation by distance (IBD) model of genetic differentiation was tested by comparing *F_ST_* (1 - *F_ST_*) with the logarithm of geographic distances, and significance evaluated using Mantel tests with 10,000 randomizations of the data. All IBD analyses were conducted using the IBDWS ([51]; available at http://ibdws.sdsu.edu/~ibdws/).

### Mitochondrial Haplotypes

Oligonucleotide primers HC02198 5′-TAA ACT TCA GGG TGA CCA AAA AAT CA-3′ and LCO1490 5′-GGT CAA CAA ATC ATA AAG ATA TTG G-3′ [52] were used for PCR amplification of ∼650 bp mitochondrial cytochrome *c* oxidase I (*cox*1) DNA barcode region. All PCR, *Sac*I PCR-RFLP and DNA sequencing reactions were performed according to [53], except cycle sequencing using BigDye™ reactions (Applied Biosystems, Foster City, CA), which were performed at the Iowa State University DNA Sequencing and Synthesis Facility, Ames, IA where data was trimmed for PHRED scores <20. The haplotype data were submitted to GenBank under the accession numbers from KJ175700 to KJ176247.

DNA sequence data were aligned for each individual using CLUSTALX 1.8 [54]. Haplotype differentiation of sequence data was estimated among 1) host plant or 2) geographic location from which samples were collected using *φ*-statistics, which is an approximation of F-statistics, based on haplotype frequencies [46], [55]. The *φ*-statistics and AMOVA estimates were obtained using Arlequin as described previously, except the Kimura 2-parameter model was used for *φ*-statistic calculation with an empirical estimated gamma parameter  =  0.05. AMOVA was used to partition haplotype variance between 1) host plants across geographic locations (sample sites) or 2) geographic location across different host plants. Pairwise *φ*_ST_ estimates were made between host plant groups using Arlequin and significance for multiple tests within each comparison determined following application of the B-Y method [46] as described above.

## Results

### Microsatellite Genotypes

The observed heterozygosity (*H*_O_) across all loci ranged from 0.02 to 0.89 while the expected heterozygosity ranged from 0.02 to 0.67. Nineteen of the 55 exact tests across host plants and geographic locations showed significant deviation from HWE. Two of the markers were monomorphic (CO241 on *L. sericeus* at Zou-Collines and 7_02K026 on *P. phaseoloides* at Ouémé-Plateau) (Table S1). MicroChecker analysis indicated that markers 7_02K06 and C0241 showed evidence of null allele presence in all populations that were tested. There was no evidence of stuttering or allele drop out in any of the microsatellite markers. Results of genetic differentiation estimates among *M*. *vitrata* were based on four sets of analyses; *analysis 1*: variation among host plants (pooled across all locations), *analysis 2*: variation among geographic locations (pooled for all host plants), *analysis 3*: differentiation between host plants within each geographic location, and *analysis 4*: differentiation between geographic locations for each host plant group.

#### Analysis 1

When microsatellite genotypes were divided into four groups based on the host plants from which *M. vitrata* larvae were collected, the global estimates of subpopulation differentiation across all loci were low but significant based on uncorrected (*F_ST_* = 0.06) and ENA-corrected microsatellite genotype data (*F_ST_* ^ENA^ = 0.05; Table 2). AMOVA results indicated that 93.03% of the genetic variation for *M*. *vitrata* was within host plant group while 5.71% was estimated among host plants (remaining data not shown). Pairwise *F_ST_* estimates of host plant differentiation based on uncorrected and ENA-corrected microsatellite data across all loci ranged from 0.01 to 0.09 (Table 3), and indicated that all comparisons were significant. *Analysis 2*: In comparison, microsatellite genotypes based on geographic location resulted in uncorrected *F_ST_* estimates of 0.02 (*F_ST_* ^ENA^ = 0.02; Table 2). Additionally, Mantel tests showed an absence of IBD through no detectable correlation between genetic and geographic distances (*R^2^* = −0.12, *P* = 0.49; remaining results not shown). *Analysis 3*: Analysis of host plant variation with a single location effectively removed a potential confounding influence of geographic variation on host plant differentiation. Subsequent pairwise *F*_ST_ estimates ranged from −0.01 to 0.28, and significant differentiation was predicted for 11 of 15 comparisons at B-Y Method adjusted significant thresholds (Table 4). Analogously, *Analysis 4* evaluated variation between geographic locations for *M. vitrata* collected from the same host plant, which predicted significant differentiation in 5 of 10 comparisons at B-Y adjusted significant thresholds (Table 5).

**Table 2:** Global and locus-by-locus estimates of subpopulation differentiation using uncorrected (*F*_ST_) and ENA-corrected microsatellite genotype data (*F*_ST_^ENA^) between four host plant groups (*V. unguiculata*, *P. phaseoloides*, *L. sericeus*, and *T. candida*) or geographic location in Benin (Ouémé-Plateau, Zou-Collines and Mono-Couffo).

Locus	Host plant groups		Geographic location	
	*F*_ST_	*F*_ST_^ENA^	*F*_ST_	*F*_ST_^ENA^
Global	0.056	0.054	0.016	0.024
C0241	0.003	0.013	0.002	0.018
7_02K06	0.109	0.111	0.055	0.077
01_B12	0.123	0.111	0.012	0.019
C32008	0.011	0.011	0.006	0.005
C0444	−0.001	0.002	−0.002	0.002

**Table 3:** Pairwise estimates of subpopulation differentiation across all microsatellite loci with and without ENA-correction (*F_ST_*) (below diagonal) and significance of corresponding comparisons (*P*-values) as indicated above the diagonal.

	*V. unguiculata L. sericeus T. candidaP. phaseoloides*				*V. unguiculata L. sericeus T. candida P. phaseoloides*			
	Uncorrected				Corrected			
***V. unguiculata***	-	0.001*	0.010*	<0.001*	-	0.001*	<0.001*	<0.001*
***L. sericeus***	0.09	-	<0.001*	<0.001*	0.13	-	<0.001*	<0.001*
***T. candida***	0.02	0.03	-	0.010*	0.01	0.09	-	0.010*
***P. phaseoloides***	0.04	0.03	0.01	-	0.04	0.13	0.01	-

**Table 4:** Estimates of *Maruca vitrata* subpopulation differentiation from pairwise *F*_ST_ between host plant groups at each geographic location (below diagonal) and significance of corresponding comparisons (P-values) as indicated above the diagonal (Oueme-Plateau, B-Y corrected α = 0.020; Zou-Collines, B-Y corrected α = 0.020; Mono-Couffo, B-Y corrected α = 0.027).

	Oueme-Plateau				Zou-Collines				Mono-Couffo			
	*V. unguiculata*	*L. sericeus*	*T. candida*	*P. phaseoloides*	*V. unguiculata*	*L. sericeus*	*T. candida*	*P. phaseoloides*	*V. unguiculata*	*L. sericeus*	*T. candida*	*P. phaseoloides*
***V. unguiculata***	–	<0.001*	<0.001*	<0.001*	–	0.85	0.285	0.082*	–	NA	0.010*	0.017*
***L. sericeus***	0.277	–	<0.001*	<0.001*	0.074	–	<0.001*	<0.001*	NA	–	NA	NA
***T. candida***	0.196	0.063	–	<0.003*	−0.005	0.056	–	0.301	0.011	NA	–	0.055
***P. phaseoloides***	0.22	0.172	0.028	–	0.001	0.1	0.001	–	0.005	NA	0.013	–

**Table 5:** Estimates of *Maruca vitrata* subpopulation differentiation from pairwise *F*_ST_ between locations from the same host plant (below diagonal) and significance of corresponding comparisons (P-values) as indicated above the diagonal (*V. unguiculata*, B-Y corrected α = 0.027; *L. sericeus*, B-Y corrected α = 0.05; *T. candida*, B-Y corrected α = 0.027; *P. phaseoloides*, B-Y corrected α = 0.027).

	*V. unguiculata*			*L. sericeus*			*T. candida*			*P. phaseoloides*		
	Oueme-Plateau	Zou-Collines	Mono-Couffo	Oueme-Plateau	Zou-Collines	Mono-Couffo	Oueme-Plateau	Zou-Collines	Mono-Couffo	Oueme-Plateau	Zou-Collines	Mono-Couffo
**Oueme-Plateau**	–	<0.001*	<0.001*	–	0.608	NA	–	0.604	<0.001*	–	0.141	0.004*
**Zou-Collines**	0.157	–	0.082	-0.006	–	NA	−0.003	–	<0.001*	0.005	–	0.219
**Mono-Couffo**	0.084	0.01	–	NA	NA	–	0.072	0.046	–	0.023	0.004	–

**Figure 2:**
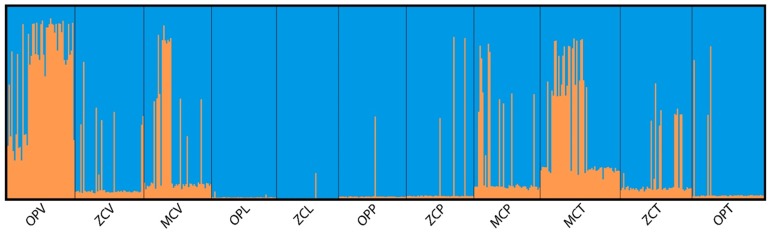
Partitioned co-ancestries among microsatellite-defined *M. vitrata* genotypes generated using the program STURUTURE with the LOCPRIOR command. For each, the estimated co-ancestry was derived from the Q-matrix for each individual and represented as vertical lines showing the proportion of the *K* = 2 segments that made up the individual genotype. Genotypes identified from the host plants *V. unguiculata*, *L. sericeus*, *P. phaseoloides* and *T. candida* across the locations are defined [OPV – Ouémé-Plateau (*V. unguiculata*), ZCV – Zou-Collines (*V. unguiculata*), MCV – Mono-Couffo (*V. unguiculata*), OPL – Ouémé-Plateau (*L. sericeus*), ZCL – Zou- Collines (*L. sericeus*), OPP – Ouémé-Plateau (*P. phaseoloides*), ZCP – Zou-Collines (*P. phaseoloides*), MCP – Mono-Couffo (*P. phaseoloides*), MCT – Mono-Couffo (*T. candida*), ZCT – Zou-Collines (*T. candida*) and OPT – Ouémé-Plateau (*T. candida*)].

STRUCTURE analysis indicated that there were 2 populations among all the samples on the different host plants and across locations (Figure 2). A maximum value of 7.41 was generated for mL”(*K*)/sL(*K*) at *K* = 2, which represented the “real” population number (*K*) that STRUCTURE predicted from microsatellite dataset. The estimated co-ancestries were partitioned into these two distinct clusters among the *M. vitrata* microsatellite genotypes, and were partitioned among host plant groups from 3 geographic locations (Figure 2). Cluster 1 (orange) was proportionately most common among *M. vitrata* samples from *V. unguiculata* at Ouémé-Plateau and Mono-Couffo, Benin as well as from *T. candida* at Mono-Couffo, Benin.

### Mitochondrial Haplotypes

The mitochondrial *cox*1 gene fragment that was PCR amplified in this study was also previously used to investigate haplotype variation among *M. vitrata* in West Africa by [53]. Alignment of novel *cox*I sequence data from 548 individuals collected from 4 different host plants at 3 different geographic locations resulted in a 619 bp consensus sequence which showed a mean nucleotide diversity of 0.0019±0.0014 (mean number of pairwise sequence differences 1.17±0.76). Results of AMOVA showed that 94.15% of the haplotype variation was within populations based on host plant from which larvae were collected, whereas 4.80% of the variation was among populations (remaining data not shown). A global estimate of haplotype differentiation among host plant groups was also low (*φ_ST_ = *0.05) but significant (*P*<0.001). Pairwise *φ_ST_* estimates which was analogous to analyses 1 to 4 used for microsatellite data (see previous section), ranged from −0.01 to 0.20 (Table 6), and showed significant differentiation for 11 of 55 comparisons at the B-Y adjusted significance threshold of 0.01 (Table S1). For example, these results showed significant variation between *φ_ST_* estimates between *T. candida* and both *V. unguiculata* and *L. sericeus* at Ouémé-Plateau, Benin. Also, *M*. *vitrata* collected from *V. unguiculata* at Ouémé-Plateau, Zou Collines and Mono Couffo, Benin showed no significant mitochondrial haplotype variation (*P*≥0.148), but *M. vitrata* collected from *T. candida* showed significant variation between all 3 geographic locations (*P*≤0.002).

**Table 6:** Pairwise estimates of mitochondrial *cox*1 haplotype differentiation among *Maruca vitrata* collected from different host plants (*φ_ST_*) (below diagonal) and significance of corresponding comparisons (P-values) as indicated above the diagonal. Significance determined at a B-Y adjusted significance threshold of α ≤ 0.020.

	*V. unguiculata*	*L. sericeus*	*T. candida*	*P. phaseoloides*
***V. unguiculata***	–	0.010*	<0.001*	0.020*
***L. sericeus***	0.02	–	<0.001*	<0.001*
***T. candida***	0.02	0.01	–	<0.001*
***P. phaseoloides***	0.01	0.04	0.06	–

## Discussion

Microsatellite markers developed from species of Lepidoptera can have high frequencies of non-PCR amplifying “null” alleles that potentially result in the overestimation of homozygosity, and have been reported in population genetic studies from a range of taxa [56], [57]. Microsatellite markers from Lepidopteran insects and molluscs have been reported to have particularly high frequencies of null alleles (review in [41]). Associations between null alleles and highly variable flanking regions have been repeatedly demonstrated (see [41]). Recent evidence suggests that null alleles at some microsatellite loci may be affected by movement of transposable elements [58]. Indeed, two of the microsatellite loci (7_02K06 and C0241) showed the presence of null alleles, but the molecular basis for the non-PCR amplification of alleles was not investigated. Regardless of the cause, resulting *F_ST_* estimates from this study were corrected using the ENA algorithm, which has previously been shown to allow for accurate analysis of population genetic microsatellite data. Both ENA-corrected as well as uncorrected *F_ST_* estimates from microsatellite data analyses provided congruent results that suggested significant levels of genetic variation exist between *M. vitrata* collected from the different host plants, but this variation is not consistently present among comparisons at different geographic locations.

Larval *M. vitrata* are a major pest of cultivated cowpea, *V. unguiculata*, in the tropics and subtropics, and are difficult to control through applications of chemical insecticides because sprays cannot contact larvae that have burrowed into the flowers and pods. The development and implementation of cowpea that expresses the *Bt* Cry1Ab toxin holds the promise to effectively control *M. vitrata* feeding damage, but the evolution of resistance in several species of Lepidoptera to *Bt* toxin has also raised concerns regarding the longevity of this technology [59]. Prior to release of cowpea varieties to farmers in West Africa, an understanding of the biology, ecology and population structure is fundamental in making sound and effective IRM decisions, which may prolong the field efficacy of this *Bt* technology. Significant levels of genetic differentiation were previously estimated among *M. vitrata* collected from *V. unguiculata* in the West African countries of Niger, Nigeria and Burkina Faso using data from SNPs [53] and microsatellite markers [38]. Genetic differentiation among *M. vitrata* populations was positively correlated with geographic distance [53]. Additionally, mitochondrial haplotypes were previously shown to be differentiated among *M*. *vitrata* collected from cowpea in the West African nations of Nigeria, Niger and Burkina Faso, with 2 distinct haplotype groups being predicted [53]. Winged insects that are capable of long distance flight (reviewed by [60]) are typically genetically homogenous [61]–[63], where admixture effectively results in a single random mating population that lacks any significant gene flow barriers [64]. *M. vitrata* persist in southern coastal repositories during the dry season and undergo a seasonal range expansion as the population migrates to northern regions when climatic conditions become more favorable at the onset of the rainy season [12], [65]. This pattern of seasonal migration may cause genetic structuring due to the Wahlund effect or other unknown population genetic factors [53], [38], but the influence of a number of other potential confounding factors was not previously investigated.

IRM programs for *Bt*-cowpea in West Africa will likely use a high-dose/refuge strategy, where refugia of non-transgenic plants will be essential for maintaining a reservoir of susceptible alleles. The high-dose/refuge strategy is considered central to managing resistance to *Bt* toxins, but the level of gene flow and random mating within and between populations of target insects is also important for the spread of susceptible genotypes in the population [22], [66]. Refugia can be comprised of cultivated non-transgenic crop plants or perhaps any other host plants that can support significant population sizes for the targeted insect pest species. Weedy species that are alternative hosts to arthropod pests may also serve as an effective form of refugia. Models based on studies of maize cropping systems suggest that increased habitat diversity, including weedy vegetation, could reduce the rate of spread of rotation-resistant western corn rootworm [67]. Studies have also reported that the utilization of wild host plants can be effective refuges within IRM strategies for transgenic crops [23]–[27]. Although *M*. *vitrata* are known to feed on multiple non-cowpea plants, the level of gene flow between individuals feeding on cowpea and these other plants remains unknown, and may affect the efficacy of IRM strategies. Many species of Lepidoptera are polyphagous and are opportunistic insects that feed on multiple alternative host plants, but instances of differential rates of development are proposed to result in reduced gene flow due to temporal variation in adult mating periods, such that assortative or structured mating systems have evolved [68]–[69]. Breakdown of gene flow between sympatric populations of a species has been hypothesized to cause host race formation [8].

Low but significant levels of genetic differentiation was estimated from microsatellite marker and mitochondrial haplotype data between *M. vitrata* collected from cultivated cowpea (*V. unguiculata*) and alternative native host plants *P. phaseoloides*, *L. sericeus* and *T. candida*. Analogous sampling of *M. vitrata* from alternative hosts was not conducted in previous studies [53] [38], and provided new insights into possible genetic structure in West Africa. Results of the current study might suggest little host plant-related *M*. *vitrata* population structure from initial analyses of microsatellite (*F*_ST_ = 0.05) and haplotype data (*φ_ST_* = 0.04). Also in contrast to previous results by [53] and [38], genetic variation in this current study was shown to be low between the 3 collection sites and not correlated with geographic distance. This might be due to our sampling that was restricted to just the southern region of Benin. Additional analyses which potentially removed the confounding influence of geographic variance showed significant pairwise genetic differentiation between *M*. *vitrata* collected from all of the different host plants at Ouémé-Plateau, but this pattern was not consistent at the Zou-Collines or Mono-Couffo locations. Similar inconsistent results were observed among pairwise comparisons of *M. vitrata* from different geographic locations but collected from the same host plant. These findings were supported by analysis with the program STRUCTURE, where co-ancestry represented by Cluster 2 (blue) was prevalent among *M*. *vitrata* collected from all different host plants, with the exception of individuals collected from *V. unguiculata* at Ouémé-Plateau and *T. candida* at Mono-Couffo.

With respect to the high dose-refuge strategy, the apparently weak and inconsistent genetic differentiation of *M. vitrata* on different host plants might suggest that high levels of gene flow would occur between susceptible individuals on wild alternative hosts and rare resistant individuals that survive on *Bt*-cowpea. Although not conclusive, our findings might also suggest that the wild hosts surveyed in this study may serve as effective refuge plants in any eventual implementation of *Bt*-cowpea in West Africa. Lack of consistent host plant differentiation among *M. vitrata* across multiple geographic locations might also suggest that the females have not become “tuned” for oviposition on specific host plants, such that host-races are not likely to have formed. More likely, complex temporal interactions between plant phenologies and attraction of female *M*. *vitrata* for oviposition may play a role in determining host plant usage and subsequent levels of gene flow at a specific locality in a specific year. Thus variation in local environments could influence oviposition and/or subsequent larval development on host plants, such that random and significant perturbations on genetic distribution might be detected. Alternatively, climatic conditions have been shown to support basal insect population sizes during conditions previously thought to be restrictive [70], such that some alternative non-cultivated hosts might harbor reservoirs of *M. vitrata* during the dry season. Sampling of these presumable small reservoir populations in this study might have inadvertently skewed our estimates of within population differentiation, and could complicate any future population genetic studies where these confounding factors are not taken into account. Regardless, our data might not suggest that random mating will occur between rare resistant moths emerging from *Bt*-cowpea and susceptible moths derived from non *Bt*-cowpea or native host plant refuges. The rate of development among *Bt* resistant individuals has been documented, such that assortative mating might be possible due to temporal delay in emergence of subsequent adults. In such a scenario, the mating period of reproductive adults may show limited overlap and could result in reduced gene flow. Under the assumptions of the high-dose/refuge strategy, temporal delays between adult emergence from *Bt*-cowpea, non *Bt*-cowpea and alternative host plants will affect the probabilities at which the rare resistant individuals mate with susceptible adults, and could lead to the rapid increase in homozygous resistant genotypes within the pest insect population if significant temporal delays are encountered.

The interactions between insect pests, their natural enemies, and the natural vegetation often leads to more efficient biological control, not only because of the increased availability of refugia and alternative prey for natural enemies during off-seasons, but also because of the higher diversity in the natural vegetation (e.g. [71]–[72]). [73] reported that the availability of alternative host plants positively affects parasitism rates, and should consequently reduce overall pest densities. Because of the semi-migratory habit of *M. vitrata*, [74] suggested two different levels from which to consider possible biological control interventions. The first option during the cropping season in cowpea fields, would be the inundative release of locally available, mass-reared trichogrammatids, preferably in conjunction with the use of pheromone trap-derived thresholds [75], particularly in areas where *M. vitrata* does not have suitable alternative host plants during the dry season, but rather invades the cowpea fields like a migrant pest (e.g., coming from the south, as it is the case for the Kano region, see [12]). The second option would be more appropriate in areas where alternative host plants are abundant and constitute a major factor influencing the dynamics of *M. vitrata* populations. In this case, inoculative releases of larval parasitoids such as *Therophilus javanus* or *T. marucae* (Hymenoptera: Braconidae) will be targeting *M. vitrata* populations on those host plants, with the objective of reducing overall pod borer populations at the landscape level. Based on the results obtained in the present study, the second option would seem more appropriate in the introduction and release of biocontrol agents against *M. vitrata*.

## Supporting Information

Table S1 **Characteristics of the** ***M. vitrata*** **individuals showing number of alleles (** ***N*** **a), number of effective alleles (** ***Ne*** **), observed heterozygosity (** ***H*** **_O_), expected heterozygosity (** ***H*** **_E_), fixation index (** ***F*** **_IS_) and probability per sample site.**
(DOCX)
