# Supplementary material for: Genetic Differentiation among Maruca vitrata F. (Lepidoptera: Crambidae) Populations on Cultivated Cowpea and Wild Host Plants: Implications for Insect Resistance Management and Biological Control Strategies
Source: PLoS One. 2014 Mar 19;9(3):e92072. doi: 10.1371/journal.pone.0092072 (PMC3960178; doi:10.1371/journal.pone.0092072)
Supplement: Table S1 — Characteristics of the M. vitrata individuals showing number of alleles ( N a), number of effective alleles ( Ne ), observed heterozygosity ( H O), expected heterozygosity ( H E), fixation index ( F IS) and probability per sample site. (DOCX) [file pone.0092072.s001.docx]

**SUPPLEMENTARY TABLE**

Table S1.

|  |  |  |  |  |  |  |  |  |
| --- | --- | --- | --- | --- | --- | --- | --- | --- |
|  |  |  |  |  |  |  |  |  |
| **Population** | **Locus** | **Na** | **Ne** | **Ho** | **He** | ***F*_IS_** | **Probability** | **Significance** |
| **Oueme-Plateau (*V. unguiculata*)** | **C0241** | 5 | 1.16 | 0.10 | 0.14 | 0.25 | 0.02 | * |
|  | **7_02K06** | 2 | 1.93 | 0.33 | 0.48 | 0.32 | 0.02 | * |
|  | **01_B12** | 2 | 1.35 | 0.22 | 0.26 | 0.13 | 0.35 | Ns |
|  | **32008** | 6 | 2.20 | 0.69 | 0.54 | -0.27 | 0.31 | Ns |
|  | **C0444** | 2 | 1.08 | 0.08 | 0.08 | -0.04 | 0.77 | Ns |
| **Zou-Collines (*V. unguiculata*)** | **C0241** | 5 | 1.29 | 0.16 | 0.22 | 0.27 | 0.20 | Ns |
|  | **7_02K06** | 2 | 1.15 | 0.14 | 0.13 | -0.08 | 0.59 | Ns |
|  | **01_B12** | 2 | 1.52 | 0.44 | 0.34 | -0.28 | 0.05 | * |
|  | **32008** | 5 | 3.00 | 0.80 | 0.67 | -0.19 | 0.43 | Ns |
|  | **C0444** | 2 | 1.08 | 0.08 | 0.08 | -0.04 | 0.77 | Ns |
| **Mono-Couffo (*V. unguiculata*)** | **C0241** | 3 | 1.09 | 0.08 | 0.08 | -0.03 | 0.99 | Ns |
|  | **7_02K06** | 2 | 1.51 | 0.10 | 0.34 | 0.70 | 0.00 | *** |
|  | **01_B12** | 2 | 1.56 | 0.43 | 0.36 | -0.19 | 0.18 | Ns |
|  | **32008** | 6 | 2.66 | 0.65 | 0.62 | -0.05 | 0.00 | *** |
|  | **C0444** | 2 | 1.14 | 0.13 | 0.12 | -0.07 | 0.64 | Ns |
| **Oueme-Plateau (*L. sericeus*)** | **C0241** | 4 | 1.15 | 0.09 | 0.13 | 0.30 | 0.00 | *** |
|  | **7_02K06** | 2 | 1.02 | 0.02 | 0.02 | -0.01 | 0.94 | Ns |
|  | **01_B12** | 2 | 1.97 | 0.37 | 0.49 | 0.25 | 0.14 | Ns |
|  | **32008** | 3 | 2.37 | 0.75 | 0.58 | -0.30 | 0.01 | ** |
|  | **C0444** | 2 | 1.02 | 0.02 | 0.02 | -0.01 | 0.94 | Ns |
| **Zou-Collines (*L. sericeus*)** | **C0241** | 1 | Monomorphic | | | | | |
|  | **7_02K06** | 2 | 1.02 | 0.02 | 0.02 | -0.01 | 0.94 | Ns |
|  | **01_B12** | 2 | 2.00 | 0.40 | 0.50 | 0.21 | 0.17 | Ns |
|  | **32008** | 4 | 2.25 | 0.89 | 0.56 | -0.60 | 0.00 | *** |
|  | **C0444** | 2 | 1.09 | 0.09 | 0.08 | -0.05 | 0.76 | Ns |
| **Oueme-Plateau (*P. phaseoloides*)** | **C0241** | 4 | 1.09 | 0.08 | 0.08 | -0.03 | 1.00 | Ns |
|  | **7_02K06** | 1 | Monomorphic | | | | | |
|  | **01_B12** | 2 | 1.16 | 0.11 | 0.14 | 0.23 | 0.12 | Ns |
|  | **32008** | 5 | 2.82 | 0.71 | 0.65 | -0.11 | 0.00 | *** |
|  | **C0444** | 2 | 1.13 | 0.13 | 0.12 | -0.07 | 0.64 | Ns |
| **Zou-Collines (*P. phaseoloides*)** | **C0241** | 2 | 1.08 | 0.08 | 0.08 | -0.04 | 0.77 | Ns |
|  | **7_02K06** | 2 | 1.11 | 0.02 | 0.10 | 0.79 | 0.00 | *** |
|  | **01_B12** | 2 | 1.30 | 0.18 | 0.23 | 0.23 | 0.12 | Ns |
|  | **32008** | 4 | 2.45 | 0.75 | 0.59 | -0.27 | 0.31 | Ns |
|  | **C0444** | 2 | 1.02 | 0.02 | 0.02 | -0.01 | 0.94 | Ns |
| **Mono-Couffo (*P. phaseoloides*)** | **C0241** | 3 | 1.04 | 0.04 | 0.04 | -0.02 | 1.00 | Ns |
|  | **7_02K06** | 2 | 1.34 | 0.13 | 0.25 | 0.50 | 0.00 | *** |
|  | **01_B12** | 2 | 1.26 | 0.19 | 0.21 | 0.07 | 0.61 | Ns |
|  | **32008** | 4 | 2.17 | 0.77 | 0.54 | -0.43 | 0.03 | * |
|  | **C0444** | 2 | 1.09 | 0.04 | 0.08 | 0.48 | 0.00 | *** |
| **Mono-Couffo (*T. candida*)** | **C0241** | 2 | 1.04 | 0.04 | 0.03 | -0.02 | 0.89 | Ns |
|  | **7_02K06** | 2 | 1.75 | 0.21 | 0.43 | 0.52 | 0.00 | *** |
|  | **01_B12** | 2 | 1.27 | 0.18 | 0.22 | 0.19 | 0.16 | Ns |
|  | **32008** | 4 | 2.30 | 0.67 | 0.57 | -0.18 | 0.00 | *** |
|  | **C0444** | 2 | 1.02 | 0.02 | 0.02 | -0.01 | 0.95 | Ns |
| **Zou-Collines (*T. candida*)** | **C0241** | 4 | 1.19 | 0.10 | 0.16 | 0.41 | 0.00 | *** |
|  | **7_02K06** | 2 | 1.19 | 0.18 | 0.16 | -0.10 | 0.49 | Ns |
|  | **01_B12** | 2 | 1.49 | 0.29 | 0.33 | 0.12 | 0.42 | Ns |
|  | **32008** | 5 | 2.84 | 0.79 | 0.65 | -0.22 | 0.00 | ** |
|  | **C0444** | 2 | 1.14 | 0.13 | 0.13 | -0.07 | 0.60 | Ns |
| **Oueme-Plateau (*T. candida*)** | **C0241** | 2 | 1.04 | 0.04 | 0.04 | -0.02 | 0.89 | Ns |
|  | **7_02K06** | 2 | 1.10 | 0.02 | 0.09 | 0.79 | 0.00 | *** |
|  | **01_B12** | 2 | 1.60 | 0.29 | 0.38 | 0.22 | 0.12 | Ns |
|  | **32008** | 4 | 2.73 | 0.82 | 0.63 | -0.29 | 0.02 | * |
|  | **C0444** | 2 | 1.02 | 0.02 | 0.02 | -0.01 | 0.94 | Ns |
|  |  |  |  |  |  |  |  |  |
